# Morphological evolution and functional consequences of giantism in tyrannosauroid dinosaurs

**DOI:** 10.1016/j.isci.2024.110679

**Published:** 2024-08-05

**Authors:** Andre J. Rowe, Emily J. Rayfield

**Affiliations:** 1 School of Earth Sciences, University of Bristol, Bristol, UK

**Keywords:** Evolutionary biology, Natural sciences, Paleobiology

## Abstract

Tyrannosauroids are a clade of theropod dinosaur taxa that varied greatly in their body size distribution. We investigated the feeding performance of six tyrannosaur genera of variable body size and skull morphology. We used 3D finite element analysis to test whether skull shape becomes more or less resistant to feeding-induced forces. Cranial and mandibular models were scaled by adult *Tyrannosaurus*’s surface area to analyze the influence of shape on skull function. It was found that *Tyrannosaurus* experienced higher absolute stresses compared to small-bodied relatives. When surface area values were equalized across genera to account for the effect of size and test efficiency of skull shape, smaller individuals experience notably greater stresses than larger relatives due to the robust cranial osteology characterized in the allometry of tyrannosaurids. These results may indicate that the wide crania of tyrannosaurids convey a functional advantage that basal tyrannosauroids, juvenile tyrannosauroids, and alioramins lacked.

## Highlights

• Giant tyrannosaurs generate consistent high bite forces but experience high stress
• At equivalent size, large tyrannosauroids evolved stronger skulls than alioramins
• Large tyrannosauroid skulls operate closer to the risk of structural failure
• Puncture-pull feeding is unlikely in alioramins and small tyrannosaurids

## Graphical abstract

**Figure:**
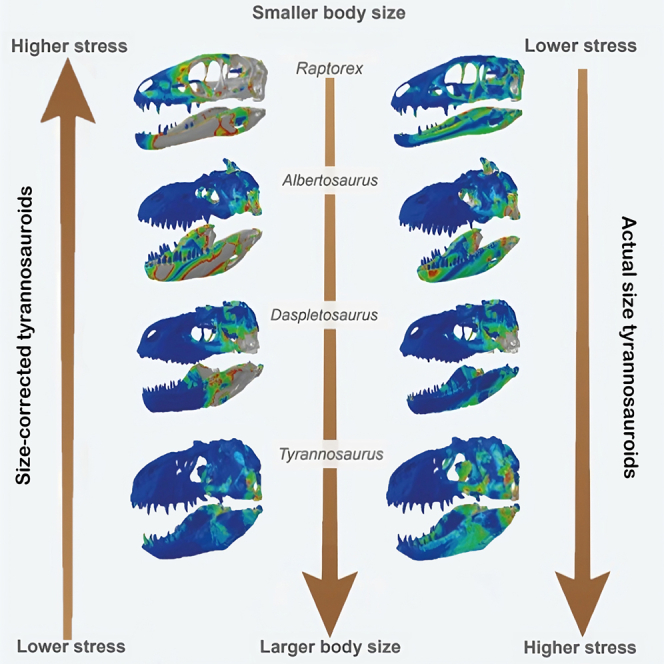

## Introduction

The theropod dinosaur clade Tyrannosauroidea contains some of the largest bipedal predators to have ever existed, including the iconic North American *Tyrannosaurus rex*,^1^^,^^2^ *Daspletosaurus torosus*,^3^ and *Albertosaurus sarcophagus*.^1^ Tyrannosauroid genera are typically recognized for their broad skulls, small forelimbs, heterodont dentition,^4^ proportionally long legs relative to body size, and large pubic boot^5^; basal members of the clade possessed lightly constructed skulls, slender body plans, and prominent cranial ornamentation in certain taxa.^6^^,^^7^ The group was first recorded in the Oxfordian stage of Middle Jurassic Asia (160 Ma ago) and gradually spread to North America.^8^ The smallest known tyrannosauroid taxa include *Guanlong wucaii*, which attained a body length of 3–3.5 m and a body mass of 125 kg^9^, and *Dilong paradoxus*, which reached a minimum length of 1.6 m^6^. The largest taxon is the 12.3–12.4 m long hypercarnivore *T. rex*,^9^ whose mature growth stages have been documented,^10^^,^^11^ though there remains some contention concerning small-bodied individuals in the clade.^12^

**Figure 1:**
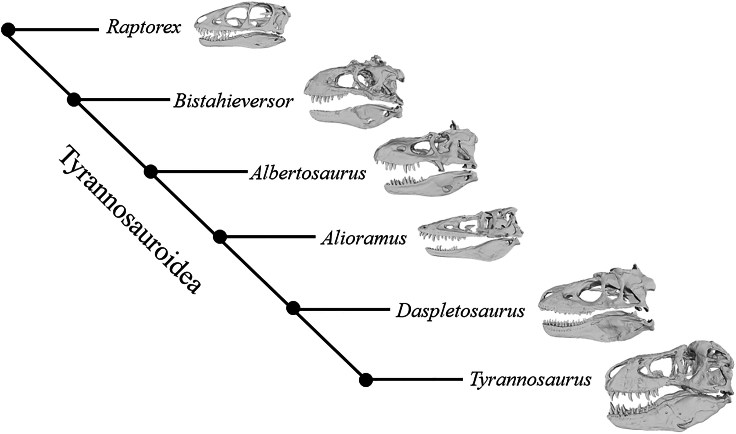
Skull models of the six genera tested plotted on a cladogram Cladogram based on Brusatte and Carr.^8^

Body size is one of the most important quantifiable properties when considering the evolution of extant and extinct metazoans as it is tied to many physiological and fitness characters.^13^ Due to the extremes of non-avian dinosaur anatomy and physiology, including their massive terrestrial body sizes,^14^^,^^15^^,^^16^ they are of particular interest in animal biomechanics.^17^^,^^18^^,^^19^^,^^20^ Theropod dinosaurs are of particular interest as they are the largest obligate bipeds described by science.^14^ The effects of large body size on locomotion and agility have been frequently studied in theropod dinosaurs.^21^^,^^22^^,^^23^^,^^24^ Conversely, the effects of large body size on feeding function have been infrequently considered quantitatively.^25^ This is despite the clade being ideal for studies of giantism and its influence on skull function, given the relatively high number of complete skulls available for study^8^ (Figure 1). Additionally, the clade evolved a wide diversity of skull morphologies; *T. rex* in particular is noted for its widely set, bone-crunching mandibles^26^ which distinguishes the genus from its slenderer-bodied relatives including *Alioramus altai*.^27^ This allows us to test hypotheses of skull shape variation and its possible relationship to feeding biomechanics.

Until recently, previous studies of theropod dinosaur feeding mechanics were largely restricted to 2D finite element (FE) models. FEA is a nondestructive modeling technique that calculates stress and strain experienced by 2D and 3D structures when a force is applied. The FE method is commonly used in engineering and medical sciences to test for weaknesses in buildings, machines, and human bones, as it can analyze any solid structures.

FEA has seen increasingly common use in studies of vertebrate evolution, usually to study skull function.^28^^,^^29^ Many FEA studies, including those on theropod dinosaur skulls, have pertained to 2D models, as they are cheaper and simpler than 3D to construct and analyze, especially in studies examining and comparing many models.^30^^,^^31^^,^^32^ FEA studies of theropod dinosaur skulls have often been limited to 2D, mainly because of the general large skull size noted in theropod dinosaur clades and the associated difficulties obtaining 3D digital shape data.^25^^,^^28^^,^^33^ Due to the growing availability of 3D scanning methods and software, which can adequately analyze 3D models quickly, FEA studies on larger vertebrate skulls using 3D models have become more common.^34^^,^^35^^,^^36^^,^^37^^,^^38^^,^^39^ Typically, comparative studies of 3D models include only 2–4 models due to the time taken to scan material, segment bones and generate accurate 3D models, and run the analyses. Furthermore, due to the massive size of some tyrannosauroid skull material, particularly derived tyrannosaurids, it is challenging to create 3D model data of various taxa. Large size is a limiting factor in studies of skull function in giant animals, particularly non-avian dinosaurs; the computed tomography (CT) scanning of taxa including adult *Tyrannosaurus* requires costly and difficult-to-use equipment.^40^ The more widely available usage of surface scanning has made the acquisition of 3D fossil data more achievable for large specimens and museums mounts.^41^^,^^42^ Thus, it is now possible to accurately investigate a giant-bodied dinosaur clade entirely using 3D FEA. Surface scan methods can capture shape but cannot resolve internal anatomy, so have traditionally not been used for structural analysis of large fossil skulls. The relative performance of surface scanned versus CT scanned skulls of the same specimens has been tested in Rowe and Rayfield.^43^ This study revealed that while solid surface scanned models were stronger than their CT counterparts, the patterns of stress and strain were almost identical. Thus, we here compare the relative performance of CT and surface scanned skulls in the same dataset. Each specimen was either surface scanned using a handheld surface scanner or generated via CT scanning, resulting in geometrically accurate 3D skull models representing the morphological diversity of Tyrannosauroidea.

In this study we take advantage of surface scanning and CT scanning to conduct a comparative analysis of skull feeding mechanics across Tyrannosauroidea. The aim of this study was to compare the cranial and mandibular biomechanical capabilities of several tyrannosauroid genera via 3D modeling and infer how bite capabilities changed with acquisition of large body size in derived tyrannosauroid species, in addition to quantifying the significance of skull shape in resisting feeding-induced load via scaling methods.

The crania and mandibles of seven tyrannosauroid specimens were analyzed: *Raptorex kriegsteini*, *Bistahieversor sealeyi*, *Albertosaurus sarcophagus*, *Alioramaus altai*, *Daspletosaurus torosus*, and two *Tyrannosaurus rex*. These individuals were selected due to the completeness of their cranial material, their phylogenetic differences including cranial morphologies and body sizes, and availability. Differences in morphologies allows us to better understand changes in feeding stresses the animals experienced as they gradually attained larger body sizes and how they withstand larger muscle forces and whether this led to greater feeding-induced cranial and mandibular stresses at their largest body sizes.

### Hypotheses

Two hypotheses were tested with results from simulated stress and strain in 3D cranial and mandible models of a variety of tyrannosauroid specimens at various body sizes and skeletal morphologies.

#### Hypothesis 1

Larger tyrannosaurid taxa such as *Tyrannosaurus rex* and *Daspletosaurus torosus* experience higher absolute stresses and strain in their skull and mandible under simulated feeding loads due to their large size and increased muscle mass. Despite this, large tyrannosaurid taxa were able to accommodate high feeding forces due to morphological differences in the skull between large and small taxa enabling large taxa to adequately absorb high stresses with minimal chance of breakage. This was one of the conclusions noted in Rowe and Snively^44^ and Johnson-Ransom et al.^39^

#### Hypothesis 2

When muscle force values for cranial and mandibular models are equalized to account for size and test for cranium and mandible shape to accommodate feeding loads, the smaller-bodied tyrannosaur specimens (*Raptorex kriegsteini*, *Alioramus altai*, and *Albertosaurus sarcophagus*) experience higher stress and strain relative to the larger-bodied taxa (*Tyrannosaurus rex*, *Daspletosaurus torosus*, and *Bistahieversor sealeyi*) due to the more robust cranial osteology in larger taxa.

## Results

### Actual size von Mises stress

**Figure 2:**
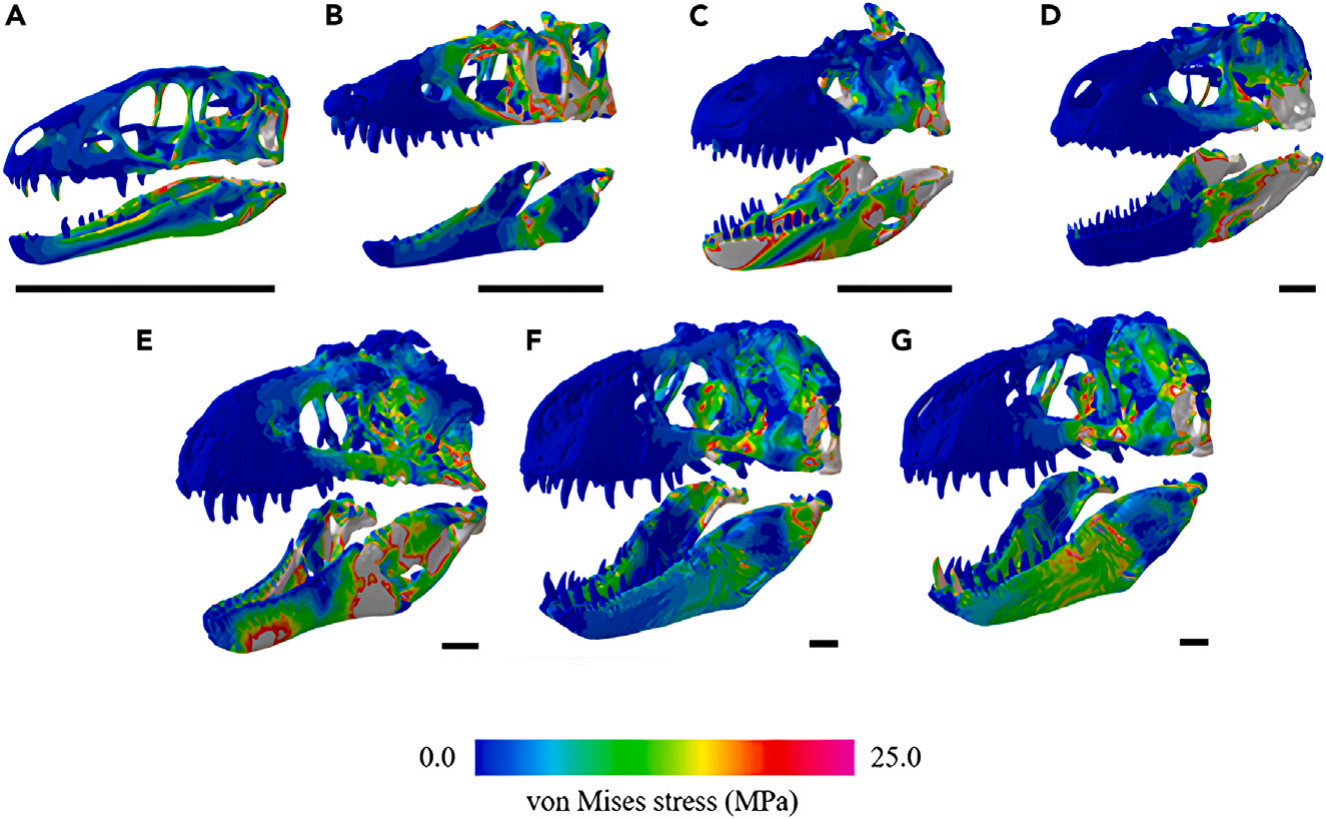
von Mises stress, a measurement of how close a structure is to breaking, for each tyrannosauroid 3D skull model tested at actual size (scale bars represent 300 mm) Warmer colors such as red and white indicate areas of high stress, i.e., where the skull is closest to breaking, while cooler colors indicate low stress. Specimens are ordered by skull length. (A) *Raptorex kriegsteini*, (B) *Alioramus altai*, (C) *Albertosaurus sarcophagus*, (D) *Daspletosaurus torosus*, (E) *Bistahieversor sealeyi*, (F) *Tyrannosaurus rex* (USNM 555000), and (G) *Tyrannosaurus rex* (FMNH PR 2081).

**Figure 3:**
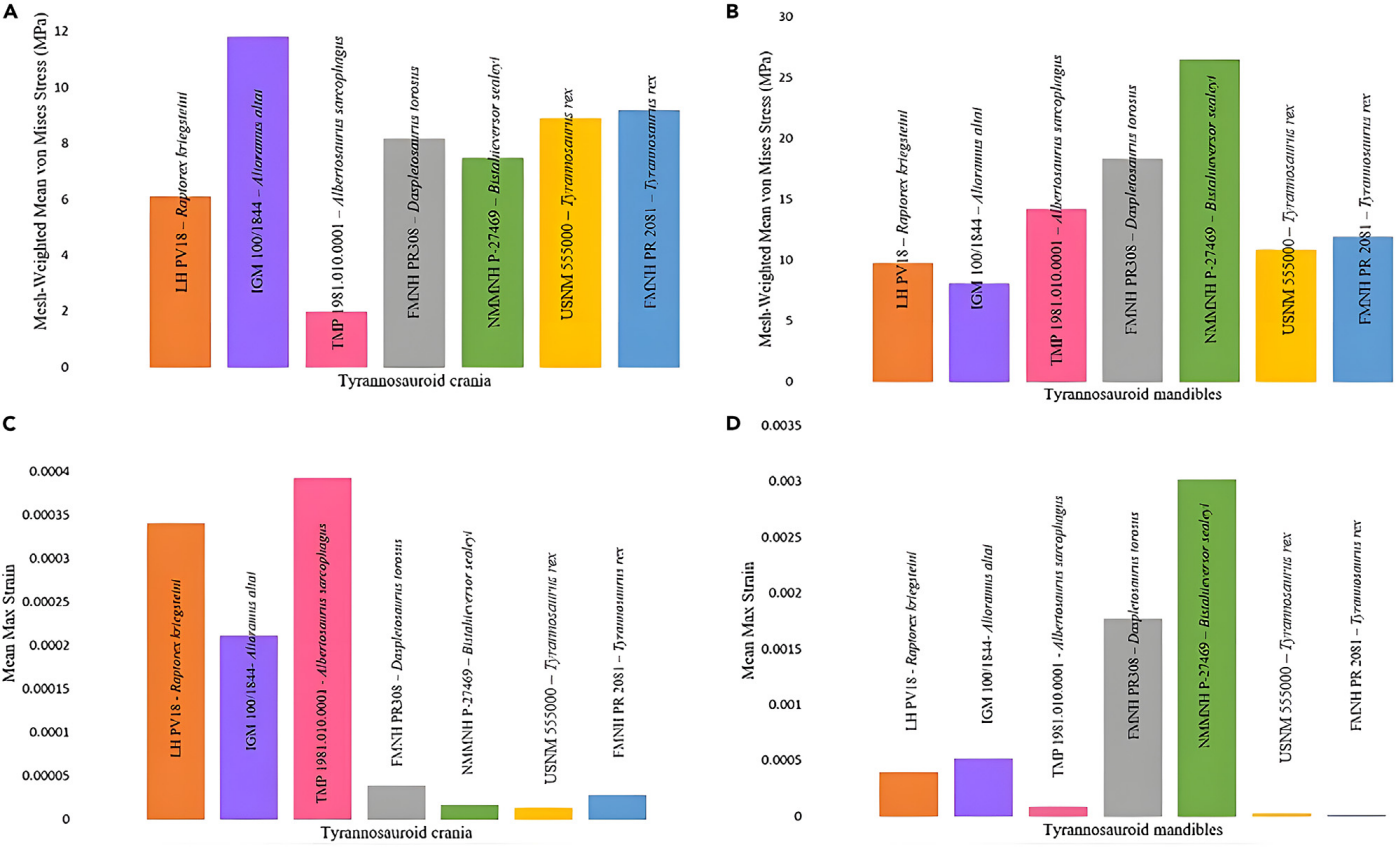
Actual size mean mesh-weighted von Mises stresses and mean maximum principle strain values for each cranium and mandible model (A) Mesh-weighted von Mises stress results for each tyrannosauroid crania. (B) Mesh-weighted von Mises stress results for each tyrannosauroid mandible. (C) Mean maximum principal strain results for tyrannosauroid crania. (D) Mean maximum principal strain results for each tyrannosauroid mandible.

We calculated the mesh-weighted von Mises stress results for tyrannosauroid crania and mandibles separately due to the complexities of analyzing an entire 3D skull model (Figure 2). When the mesh-weighted arithmetic mean (MWAM) was calculated, the overall highest von Mises stresses were experienced by the middle-sized tyrannosauroids, *Daspletosaurus torosus* and *Bistahieversor sealeyi*, particularly at the mandible (Figures 2A, 2B, and 3). Similarly high bending stresses were calculated for the two adult *Tyrannosaurus rex* specimens, the largest individuals in the dataset. The lowest bending stresses were observed in the three smallest tyrannosauroids, *Albertosaurus sarcophagus*, *Alioramus altai*, and *Raptorex kriegsteini*.

### Actual size maximum principal strain

In addition to exporting von Mises stress results from Abaqus/CAE 6.14–1, we exported and calculated the mean maximum principal strain values for each mesh (Figures 2C and 2D). These values were exported separately from the von Mises stress results and elemental volume data.

### Surface area equalized von Mises stress

**Figure 4:**
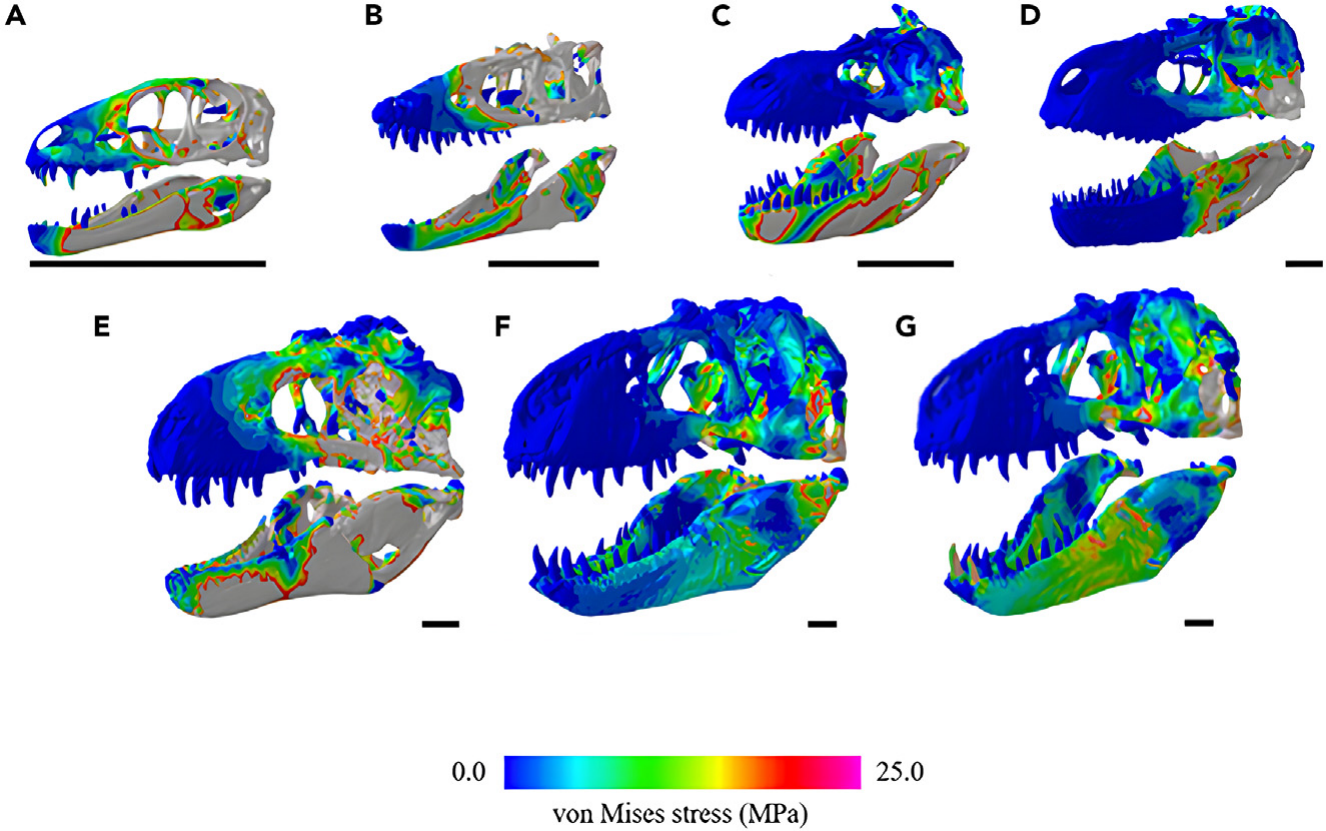
von Mises stress for each tyrannosauroid 3D skull model when surface area values were scaled to FMNH PR 2081 (scale bars represent 300 mm) Note the high bending stresses in the smaller tyrannosauroid models relative to the larger individuals as indicated by areas of red and gray, particularly in *Raptorex* and *Alioramus*, while the *T. rex* crania appears more stress resistant. (A) *Raptorex kriegsteini*, (B) *Alioramus altai*, (C) *Albertosaurus sarcophagus*, (D) *Daspletosaurus torosus*, (E) *Bistahieversor sealeyi*, (F) *Tyrannosaurus rex* (USNM 555000), and (G) *Tyrannosaurus rex* (FMNH PR 2081).

**Figure 5:**
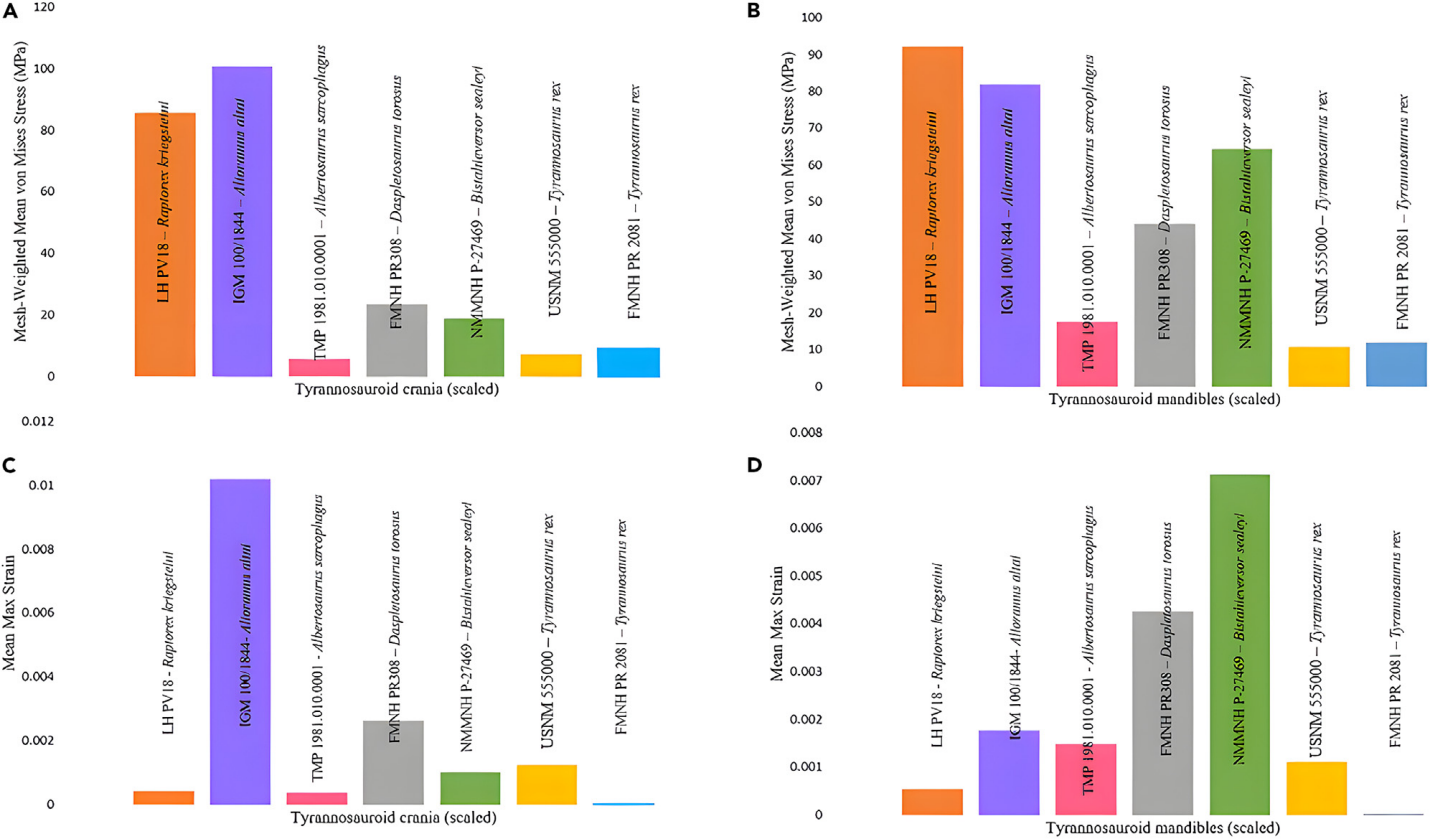
Surface area scaled mean mesh-weighted von Mises stresses and mean maximum principle strain values for each cranium and mandible model (A) Mesh-weighted von Mises stress results for the tyrannosauroid crania. (B) Surface area scaled mesh-weighted von Mises stress results for the tyrannosauroid mandibles. (C) Surface area scaled mean maximum principal strain results for the tyrannosauroid crania. *Alioramus* experienced much higher strain than the other individuals, possibly due to the extensive reconstructions applied (see discussion). (D) Surface area scaled mean maximum principal strain results for the tyrannosauroid mandibles.

When muscle force component values were scaled to FMNH PR 2081, the largest individual in the dataset, to normalize size and account for only shape, smaller tyrannosauroid specimens generally experienced much higher von Mises stresses (Figures 4 and 5). *Alioramus* was an exception in terms of cranial stresses; it experienced noticeably higher stresses than the smaller *Raptorex*, though this may be due to anatomical reconstructions (see discussion). Additionally, *Daspletosaurus* and *Bistahieversor* demonstrated particularly high stresses in their mandible models relative to the smallest tyrannosauroids.

### Surface area equalized maximum principal strain

In addition, we exported and calculated the mean maximum principal strain for each specimen when scaled to FMNH PR 2081 (Figure 5). Cranial models of smaller tyrannosauroids were the most strained in these scenarios, while the mandibular models demonstrated a less clear trend; *Daspletosaurus* and *Bistahieversor* were the most strained models. The other *Tyrannosaurus* model (USNM 555000) was the least strained for both types of models.

### Muscle sensitivity results

The mean von Mises stress values were calculated for each group of sensitivity tests per taxa (Tables 1 and 2). Specimens were chosen to represent the range of body sizes and morphologies in the specimen pool (small, medium, and large). While there is no margin of error typically associated with sensitivity testing, we were interested in quantifying any potential differences in types of models (CT or surface scan) and by model size. The greatest variability resulted from the *Albertosaurus* model, particularly at the mandible (Table 2), while the *Tyrannosaurus* and *Raptorex* models demonstrated less variability overall, though the *Raptorex* and *Albertosaurus* cranial results were comparable (Table 1).

**Table 1:** Muscle sensitivity results for three different tyrannosaurid crania tested

Tyrannosaurid crania	Baseline von Mises stress mean	von Mises stress mean 10% higher	von Mises stress mean 10% lower	Percentage von Mises stress mean difference (10% higher)	Percentage von Mises stress mean difference (10% lower)
*Raptorex kriegsteini*	7.111209	7.729046	7.748312	8.3265%	8.57501%
*Albertosaurus sarcophagus*	1.988823	1.905102	2.183277	4.30008%	9.32164%
*Tyrannosaurus rex* (FMNH PR 2081)	9.201477	9.790244	9.110124	6.20025%	0.997761%

**Table 2:** Muscle sensitivity results for three different tyrannosaurid mandibles tested

Tyrannosaurid mandibles	Baseline von Mises stress mean	von Mises stress mean 10% higher	von Mises stress mean 10% lower	Percentage von Mises stress mean difference (10% higher)	Percentage von Mises stress mean difference (10% lower)
*Raptorex kriegsteini*	9.720271	9.920344	9.901121	2.03734%	1.8434%
*Albertosaurus sarcophagus*	14.21646	17.31926	17.92455	19.678%	23.0739%
*Tyrannosaurus rex* (FMNH PR 2081)	11.9148	12.3112	13.2155	3.27252%	10.3516%

## Discussion

Our true size FE results indicate somewhat higher von Mises stresses occurring in larger tyrannosaurids, particularly in the middle-sized specimens such as *Bistahieversor* (Figures 2 and 3). This may be due to the notably deep skull of *Tyrannosaurus* acting as a buffer against the high stresses of a bone-crunching bite,^26^^,^^44^ which smaller tyrannosaurids may have been less capable of inflicting,^45^ or due to higher stresses and strain which tend to occur in 3D models that have undergone a high degree of reconstruction/retrodeformation,^43^ particularly the *Alioramus* cranium included in this study.

### Significance of large body size

Our main results indicate an advantage in terms of stress resistance during feeding in large-bodied tyrannosauroids, most notably in *Tyrannosaurus*. This was demonstrated in the scaled results, where small tyrannosauroids experienced higher bending stresses, particularly at the mandible; *Raptorex* experienced roughly nine times the stresses of *Tyrannosaurus* (Figure 5). *Tyrannosaurus* is commonly noted for its widely set jaws and deep skull^46^; the robust shape likely enables it to accommodate high stresses and effectively prey on proportionally large prey items.^47^^,^^48^ A similar phenomenon is observed in giant pliosaurs,^49^ wherein the large adults experience higher stresses than small individuals.

Slenderer tyrannosauroids such as *Qianzhousaurus sinensis* have previously been hypothesized to use their more gracile bodies to better pursue smaller prey which would not have required a powerful, puncture-pull bite to subdue.^50^ Given the close relationship between *Qianzhousaurus* and *Alioramus*, our results generally support these hypotheses, as the *Alioramus altai* skull was one of the most highly stressed during feeding scenarios in this study. Thus, we predict it would not have been capable of withstanding the stresses of a powerful bite and would have instead pursued proportionally small-bodied prey to avoid competition with the sympatric *Tarbosaurus*, which may have preyed on large hadrosaurs and titanosaur sauropods.^51^^,^^52^ Prey body size alone is not likely to be the only factor in competition avoidance between sympatric carnivores given juvenile *Tarbosaurus* would presumably achieve sizes comparable to mature alioramins. Feeding traces attributed to *Tarbosaurus* have been identified on a variety of giant herbivorous Mongolian dinosaurs including the hadrosaurid *Saurolophus*,^53^ ornithomimosaur *Deinocheirus*,^51^ and sauropod *Opisthocoelicaudia*.^54^ The lack of feeding traces attributed to alioramins and *Raptorex* makes their feeding preferences less obvious, and awaits more direct evidence from the fossil record.

The conclusions we can derive from the middle-sized tyrannosauroids, i.e., those smaller than *Tyrannosaurus* but larger than *Alioramus*, are less clear. Given their somewhat higher stresses at actual size, it is unlikely that *Daspletosaurus* and *Bistahieversor* could withstand the resultant forces associated with *Tyrannosaurus*’s exceptionally powerful bite.^26^^,^^37^ However, when using actual sized 3D models, these medium-sized tyrannosauroids did not differ significantly from either *Tyrannosaurus*, particularly at the cranium (Figure 3). Given the available data and feeding traces from tyrannosaurids such as *Daspletosaurus*^55^ and *Tyrannosaurus*,^56^^,^^57^^,^^58^ it is a fair deduction that large tyrannosauroids all functioned as generalist puncture-pull feeders as our FE results demonstrate overall comparable skull strengths in all tyrannosauroids larger than *Raptorex* and *Alioramus*. While it is most probable that all tyrannosauroids were opportunists that spent most of their time searching for food like modern taxa,^59^ a difficult question to assess is the ratio of food that is actively hunted versus scavenged, or how it varies between taxa. Middle-sized tyrannosauroids may have been reliant on different feeding strategies when compared to *Tyrannosaurus*, and further work is needed to establish this possible difference throughout the clade.^53^^,^^60^

### Ontogenetic patterns in relation to feeding mechanics

Previous work has assessed the feeding performance of juvenile tyrannosaurines when compared to a senescent individual.^39^^,^^44^ Rowe and Snively^44^ primarily quantified von Mises stresses in the mandible of *Raptorex*, a juvenile *Tyrannosaurus* (BMRP 2002.4.1), and a mature *Tyrannosaurus* (FMNH PR 2081) using CT scanned 3D models. It was found that, when tested at true size, older, larger individuals experienced higher overall feeding-induced stresses due to the widely set jaws associated with mature tyrannosaurids. When individuals were scaled to equivalent size, smaller, immature individuals experienced higher overall bending stresses.

Similarities were noted in this study, where larger tyrannosauroid skulls comprising different genera yielded comparatively higher von Mises stresses, particularly at the cranium (Figure 3) in mature *Tyrannosaurus*. Large tyrannosauroids possessed strongly constructed skulls but also experienced higher stresses than smaller taxa at actual size and are thus accommodating more force per unit area. This may represent a trade-off where the expanded tyrannosauroid posterior skull and increased adductor muscle mass generate high bite forces, which results in the need to accommodate greater stresses in the skull during feeding. The trend applies to ontogenetic stages in *Tyrannosaurus* as observed in Rowe and Snively,^44^ and appears to occur in some large tyrannosauroid taxa in our study. Ma et al.^33^ similarly noted a trend in jaw strengthening ontogenetically in tyrannosaurids using 2D models of *Tarbosaurus* and *Tyrannosaurus* and attributed bone functional adaptation to the overall mandible strengthening observed in mature individuals. Johnson-Ransom et al.^39^ noted higher jaw muscle forces in a juvenile *Tyrannosaurus* cranium when compared to other tyrannosaurine genera including *Teratophoneus.* Overall, the *Tyrannosaurus* skull is well-suited to absorbing high cranial and mandibular stresses during feeding, likely necessitated by the large-bodied prey in its environment.^61^ In particular, *Edmontosaurus* is noted for its reduction in stride length to preserve energy and run efficiently^62^; thus, the ability to ambush and quickly subdue a large animal requires both high biting forces and the accommodation of high feeding stresses throughout the skull.

### 3D scanning methodology and its influence on 3D finite element data

Because of the differences in results that arise in FE studies using both CT scans and surface scans,^43^ this necessitated the use of the MWAM. This method alleviates potential von Mises stress differences by incorporating element volume into the calculation of stress. However, even when the MWAM is calculated for 3D FEA data, extensive mesh reconstructions such as duplicating and mirroring a mandible ramus to replace a broken half are often a factor generating von Mises stress outliers, which were the steps taken when we retrodeformed *Bistahieversor*’s mandible. 3D FEA of the skull requires all bones to be present, and hence, some reconstructions will be necessary, particularly when analyzing fossil taxa that are often broken, incomplete, or deformed due to geologic processes.

The *Daspletosaurus* (surface scan), *Bistahieversor* (CT derived), and *Alioramus* (CT derived) specimens sometimes demonstrated higher stresses when compared to the other tyrannosauroids, particularly at the mandible; *Daspletosaurus* and *Bistahieversor* experienced higher stresses than the larger *Tyrannosaurus* specimens. Notably, the *Alioramus* experienced higher cranial stresses and particularly maximum principal strain (Figure 3) than the smaller *Raptorex* specimen; this trend did not occur in the mandibles, which may be due to the anatomical simplicity of the mandibles requiring less intensive reconstruction efforts. The high stresses in *Daspletosaurus* and *Bistahieversor* may be due to extensive reconstructive modeling, and the low stresses and variability in muscle sensitivity testing in *Albertosaurus* (Tables 1 and 2) may be attributed to its status as a compacted replica. However, despite reconstructions of these 3D models, our models demonstrate a clearer quantitative trend toward low stress in progressively larger, scaled individuals (Figure 5) which was more unlikely to occur if the reconstructions were influencing our FE output in a notable way.^43^ Thus, we conclude that these results may be attributed to both 3D model retrodeformation and potentially due to the slenderer, ancestral skull morphologies associated with basal tyrannosauroids.

### Future work

Many individuals in Tyrannosauroidea are amenable to CT and surface scanning than the total number included in this study, including the basal tyrannosauroid *Xiongguanlong baimoensis*^63^ and the alioramin *Qianzhousaurus sinensis*.^64^ Large, derived tyrannosaurid specimens ideal for 3D FEA include *Lythronax argestes*,^65^ *Gorgosaurus libratus*,^66^ and *Daspletosaurus horneri*,^67^ though certain individuals including *Lythronax* require some degree of reconstruction. Most of the replica specimens included in this dataset did not provide noticeable outliers in terms of skull stresses; the exception, *Albertosaurus sarcophagus*, was a more compacted replica, unlike the *Daspletosaurus* and *Raptorex*. Museum replicas generally work well in FE studies, assuming a high degree of similarity to the original fossil in cases where the original is not available.^41^ Given its favored prey of larger dinosaurs in its ecosystem,^51^^,^^52^ a large, complete *Tarbosaurus bataar* skull with minimal geologic deformation would be an excellent candidate for CT scanning and FEA, to better elucidate how it compares to *Tyrannosaurus* without the potential influence of 3D reconstructions.

### Conclusion

The main conclusions of this study are that (1) tyrannosauroid dinosaurs generally experienced higher stresses in their skulls during feeding as body size increased and (2) the widely set jaws of large tyrannosaurids enabled those taxa to better accommodate high degrees of von Mises stress during feeding. Thus, the data support both our hypotheses concerning tyrannosauroid body size and its relationship to feeding biomechanics. While large body size seems to cf. a functional advantage in tyrannosaurids, particularly *Tyrannosaurus*, smaller individuals including *Alioramus* may have utilized their differing size in an ecological niche distinct from *Tyrannosaurus* and other giant theropods. The ability to effectively absorb high feeding stresses may have contributed to the evolutionary success of large tyrannosaurids, which functioned as generalist puncture-pull feeders.

### Limitations of the study

One limitation of the present work is the influence of digital reconstructions on 3D models during FE modeling. This was particularly notable in the *Alioramus* skull, which was completely disarticulated and digitally restored using a combination Artec Studio Professional 14, Blender, and Geomagic Studio 12. Similar retrodeformation work was carried out on *Daspletosaurus* and *Bistahieversor*. Both the *Raptorex* and two *Tyrannosaurus* models were left as is; we predict that if these models required retrodeformation work, their stresses and strains would accordingly increase, though the degree to which this happens varies depending on the amount of retrodeformation applied. Additionally, internal anatomical regions of surface scanned material can be difficult to appropriately model, which is a non-issue in CT scans, which capture internal geometries of solid structures. Hence, stresses and strain in CT-derived models may be relatively higher than those of surface scanned equivalents given the infilling that tends to occur in surface scan reconstructions.

## STAR★Methods

### Key resources table

**Table:** 

REAGENT or RESOURCE	SOURCE	IDENTIFIER
**Deposited data**		

Abaqus/CAE files for tyrannosauroid FEA	Mendeley Data	https://data.mendeley.com/drafts/7sshbwc6ms

**Software and algorithms**		

Abaqus/CAE	https://www.3ds.com/products/simulia/abaqus/cae/	Abaqus/CAE Version 6.14-1
Altair HyperMesh	https://altair.com/	HyperMesh Version 2021
Artec Studio Professional	https://www.artec3d.com/3d-software/artec-studio/	Artec Studio Professional Version 14
Avizo Lite	https://www.thermofisher.com/uk/	Avizo Lite Version 9.5
Blender	Blender Foundation https://www.blender.org/	Blender Version 2.82
Geomagic Studio	https://www.3dsystems.com/software/	Geomagic Studio Version 12
MeshLab	https://www.meshlab.net/	MeshLab 2020.06
RStudio	RStudio Team https://www.rstudio.com/	RStudio Version 2023.06.1

### Resource availability

#### Lead contact

Further information and requests for resources should be directed to and will be fulfilled by the lead contact, Andre J. Rowe (andre.rowe@bristol.ac.uk).

#### Materials availability

This study did not generate new reagents.

#### Data and code availability

• Abaqus/CAE files have been deposited at Mendeley Data and will be publicly available upon publication. The DOI is listed in the key resources table.
• No original code was generated in this study.
• Any additional information required to reanalyse the data reported in this paper is available from the lead contact upon request.

### Method details

#### Specimens

In this study, seven individual tyrannosauroid skull specimens representing six genera were tested using 3D finite element analysis. As indicated in Tables S1 and S2, the taxa vary greatly in skull length, overall estimated body masses, and ontogenetic stage. Femur length was not included despite being a commonly used proxy for body size estimates due to unavailability of femoral material for some taxa.^71^

We included *Bistahieversor sealeyi* is a basal eutyrannosaurian from the Campanian (75.5-74.5 Ma) of New Mexico, USA, from the Kirtland and Fruitland Formations.^72^ It is one of the largest tyrannosauroid genera, estimated to have attained body lengths of 9 m.^73^ It is noted for its brain and sinus system which was nearly identical to tyrannosaurids like *Tyrannosaurus*,^74^ as well as possessing a deep snout, indicating that it is an anatomical characteristic not exclusive to derived tyrannosaurids.^72^ Its feeding biomechanics have not previously been analyzed using 2D or 3D FEA.

*Albertosaurus* is a genus of large-bodied tyrannosaurid from Middle Maastrichtian of northwestern North America approximately 71 Ma. The type species, *A. sarcophagus*, is included in this study.^1^ While it is one of the larger tyrannosaurid genera described in the literature, reaching lengths of 8–9 m^3^, it is still notably smaller than other Late Cretaceous tyrannosaurids such as the 4.5–5 tonne *Tarbosaurus*.^73^ *Albertosaurus* is notable for its relatively low bite forces relative to other tyrannosaurids, attaining maximum estimates of 3, 413 N for its posterior teeth^45^ compared to *Tyrannosaurus* estimates which range from 8,526 to 63,429 N.^26^^,^^37^^,^^75^ The genus may have engaged in gregarious behavior,^76^^,^^77^ as well as cannibalism.^78^

We also include *Daspletosaurus torosus* as another large-bodied tyrannosaurid from Campanian to Maastrichtian (77-75 Ma) North America. It is one of the larger tyrannosaurids, attaining lengths of 9 m^3^. *Daspletosaurus* exhibits evidence of facial biting behaviors, indicating intraspecific aggressions that were possibly for territory, resources, or dominance within social groups,^79^ though it is not exclusive to the genus.^80^ Additionally, like in *Albertosaurus*, there is evidence of cannibalism^81^^,^^82^ and social groups,^83^ though the latter is disputed.^84^

*Alioramus* is a subadult tyrannosaurid which is noted for its relatively slender body and smaller mass estimates.^27^^,^^64^ It is represented by two species, *A. remotus*^85^ and *A. altai*^27^; *A. remotus* does not have a complete skull and we therefore used *A. altai* in our analyses. It has been suggested that alioramin genera maintained their slender bodies into maturity and hunted smaller, more agile prey to avoid competition with larger tyrannosaurids such as *Tarbosaurus*.^50^ Additionally, they lack the deep maxilla and peg-like teeth present in large tyrannosaurid genera, and were therefore unlikely to employ the puncture-pull feeding technique characteristic in large genera.^27^

*Raptorex kriegsteini* is a taxon which is recognized from a single individual recovered from Mongolia. It was first considered to originate from the Barremian–Aptian of Lower Cretaceous China (∼125.8–124.1 Ma)^86^; later work placed it as Late Cretaceous and likely originating from the Nemegt of Mongolia.^87^ It is characterized by its relatively large skull, strong hindlimbs, and two-fingered forearms which are noted in tyrannosaurids, in contrast to basal tyrannosauroids.^86^ The specimen has been estimated to be roughly 5–6 years of age based on lines of arrested growth (LAGs).^87^ Due to its age and proximity to the large Mongolian tyrannosaurid genus, *Tarbosaurus*,^88^ it may represent a juvenile form; however, this has been disputed in phylogenetic analyses of tyrannosaurids and it may instead represent a valid genus.^8^^,^^89^ Its mandibular properties were analyzed using FEA in Rowe and Snively,^44^ while Johnson-Ransom et al.^39^ examined its cranial mechanics also using FEA. Rowe and Snively^44^ concluded that *Raptorex*’s mandible experienced relatively low von Mises stresses in contrast to the mature individuals, suggesting that subadult or smaller tyrannosaurid genera fed on smaller, potentially more agile prey. Johnson-Ransom et al.^39^ concluded that small early-diverging tyrannosauroids (*Dilong* and *Proceratosaurus*) exhibit muscle forces that are lower than *Raptorex*.

Lastly, we included two *Tyrannosaurus rex* specimens. USNM 555000 (formerly MOR 555) and FMNH PR 2081 are both mature specimens, though USNM 555000 is considered to be a younger adult of approximately ∼23–27 years old at death, while FMNH PR 2081 is approximately 28.^11^ FMNH PR 2081 is often noted as one of the most complete and senescent *T. rex* specimens^90^ and it is the largest specimen in this study, with a body length of 12.3–12.4 m.^91^ Many previous studies have examined feeding biomechanics and behaviors in *Tyrannosaurus*: Lautenschlager^92^ tested the maximum jaw gape in the genus to be roughly 80°, which was necessary to power the animals’ puncture-pull bite. Snively et al.^93^ determined that the fused nasals in *Tyrannosaurus* could withstand high compressional forces and strengthened the cranium in vertical bending, which was hypothesized in Rayfield.^94^ Furthermore, its powerful neck muscles would have enabled its puncture-pull feeding as well as rapid prey strikes.^95^ There is evidence of predatory behavior in the genus^48^ as well as cannibalism.^96^

We compiled skull lengths from the literature and measurements from our own scans using MeshLab 2020.06 for each specimen to quantify size differences between taxa (Table S1), as well as occipital condyle measurements which may also act as body size proxies in dinosaurs^97^ and therian mammals.^98^ Additionally, we compiled body mass estimates and ontogenetic stages for each specimen (Table S2).

#### Surface scanning

In this study, three models were constructed from surface scan datasets. We included a replica of the large North American tyrannosaurid *Daspletosaurus torosus* (FMNH PR308) which is housed in the Field Museum of Natural History Geology Collections. Due to the size and mass of the skull, the individual was surface scanned using an Artec Space Spider blue light surface scanner. The crania and mandibles were scanned as separate project files because large file sizes typically result in slowdown and occasional software crashes in Artec Studio Professional 14 during scanning.

In Artec Studio Professional 14, the scans were aligned to digitally reassemble the entire skull. Stray pixels were deleted, as well as frames with max error values above 0.3, as those at 0.4 or higher are more likely to result in errors. We then applied global registration to convert all one-frame surfaces to a single coordinate system using information on the mutual position of each surface pair. The Sharp Fusion tool was used to create a polygonal 3D model, which solidifies the captured and processed frames into an STL file type, which are commonly used in 3D modeling software. We used Sharp Fusion rather than Fast or Smooth Fusion as it best preserves fine details of scans, including small teeth and rugose bone textures which may be lost otherwise. Lastly, we used the small-object filter to clean the model of floating pixels and the ‘fix holes’ function to fill any gaps.

The *Daspletosaurus*’s cranial model had a filled antorbital fenestra, promaxillary fenestra, and nares. These elements were hollowed out in the final STL model using the 3D eraser tool in Artec Studio Professional, to be as anatomically accurate as possible (Figure S1) and consistent with other 3D models.

The *Albertosaurus sarcophagus* skull was surface scanned at the Las Vegas Natural History Museum (LVNHM) and downloaded from Sketchfab. It is a replica of a disarticulated specimen at the Royal Tyrell Museum of Paleontology which only contains the left jugal and maxilla in their original articulation (D. Henderson, personal communication 2022). The STL file of the skull model was originally comprised of both the cranium and mandible as a single model. The downloaded file was uploaded into Blender 2.82, where the mandible was deleted and the file was saved with only the cranium. A similar protocol was then followed to create a file containing only the mandible model so that both parts of the skull could be analysed separately, as fully articulated skull models are difficult to run using FEA.

#### Computed tomography scanning

Four of the seven tyrannosauroid skull specimens analysed in this study were CT scanned at various institutions in the United States. These include a senescent *Tyrannosaurus rex* (FMNH PR 2081), the *Bistahieversor sealeyi* holotype (NMMNH P-27469), *Aliormaus altai* (IGM 100/1844), and a replica of the juvenile *Raptorex kriegsteini* (LH PV18). The *Tyrannosaurus rex* was CT scanned at Rocketdyne Division, Boeing North America, Inc., of Chatsworth, CA using a Minatron 205 scanner.^90^ The Minatron 205 scanner, built by Scientific Measurement Systems, Inc. generated 748 2 mm thick coronal slices. These were manipulated with VoxBlast 2.2 (VayTek, Inc., Fairfield, IA) to make three-dimensional models and to generate synthetic resliced stacks in the sagittal and horizontal planes. Slice thickness was 2mm for the full-skull sagittal and horizontal sets, and 0.5mm for the horizontal stack through the braincase.

The *Bistahieversor* 3D model was generated from CT scanning at the Microtron Facility at Los Alamos National Laboratory, using a Bremsstrahlung source (Scanditronix M22 medical therapy source, Microtron) with 10MeV x-rays with a 0.25mm lead filter.^74^ The *Alioramus altai* (IGM 100/1844) skull bones^27^ were CT scanned in a GE phoenix v|tome|x CT scanner at the American Museum of Natural History Microscopy and Imaging Facility. 13 elements were scanned with the following general settings: voltage between 140 and 210 kV, amperage between 125 and 175 μA, and a slice thickness ranging from 0.09 to 0.14 mm.^99^ Because of the *Alioramus* skull disarticulation, we assembled the bones into a complete skull using Artec Studio Professional 14. This was done by individually opening the STL files in Artec Studio, aligning the bones based on imagery from Brusatte et al.,^27^ and exporting the combined meshes as a single file. Two STL files were generated, consisting of a separate mandible and cranium.

Because of taphonomic deformation that is commonly seen in vertebrate fossils and the influence deformation can have on data,^100^^,^^101^ the 3D model of *Bistahieversor* was retrodeformed using mesh editing software. This was achieved by importing the deformed model into Artec Studio Professional 14 and deleting the more severely deformed right half of the cranium. The lesser deformed half of the cranium was then duplicated, mirrored, and reattached to the original along the midline. The now-complete skull was uploaded into MeshLab 2020.06 to check for and delete self-intersections which are common in procedures where 3D models are merged (Figure S2). This step was vital as self-intersecting triangles make meshing impossible and will otherwise lead to errors in FEA. The model was then imported into Blender 2.82 and the ‘Sculpt’ module was selected. The midline of the model was then smoothened to better resemble how the skull would appear when the skull was undeformed so our analyses would better reflect biological reality. Lastly, the model was uploaded into Geomagic Studio 12 and the Mesh Doctor tool was selected, which corrects the model for spikes, holes, self-intersections, and other potential sources of error when meshing a 3D model.

#### 3D model meshing and finite element analysis

Models were decimated to be at varying triangle counts to best avoid overly long analysis times and freezing issues when meshing the models in HyperMesh while maintaining intricate details in the models (Table S3). The models were then imported into Geomagic Studio 12 and the Mesh Doctor tool was selected to clear the models of self-intersections, spikes, and highly creased edges. These geometric issues may cause errors when meshing in HyperMesh, and thus were critical to remove. Once the models were error-free and watertight, they were imported into HyperMesh (Altair) as four-noded tetrahedral elements, which are generally the standard in 3D FEA.

The HyperMesh output format was selected for Abaqus/CAE 6.14-1, as meshed models were saved as CAE files which are used for FEA in Abaqus. In the volume tetra sub-panel, element size was set to 10, and in the Tetramesh parameters sub-panel we selected for optimized mesh quality and for the mesh speed to be gradual, as these settings generally maximize accuracy in FE data. We then highlighted the entire model and selected the mesh tool. Once meshing was successful, we applied the appropriate material properties to the meshed models. The bone properties were assigned based on sub-adult *Alligator mississippiensis* skull bone: Young’s modulus of 15,000 MPa and Poisson’s ratio of 0.29.^102^^,^^103^^,^^104^ Crocodilian bone properties were selected because of the close evolutionary relationship between crocodilians and dinosaurs. Dentine properties were not applied to the teeth, as they do not affect general stress plot comparisons in 3D von Mises stress results.^105^

Constraints were selected for under the Load Collectors tab and then applied at the quadrate in cranial models, and at the hinges of the articular in the mandibles to prevent the models from freely floating during FEA. Three constraints were selected for each hinge of the quadrate or articular, for a total of six constraints per model. The models were constrained from rigid body motion in all directions by selecting a single degree of freedom for X, Y, and Z. We applied feeding loads by selecting the Load Collectors tab, selecting card image ‘history,’ and checking ‘CLoad’ and ‘Load Case.’ In the Forces sub-panel, five nodes were selected for each premaxillary tooth at both sides of the cranium or mandible, totalling 10 nodes for each analysis. This was to simulate feeding in the animals; in these cases, it simulates the premaxillary and anterior dentary teeth making contact with prey items. Lastly, we selected for a ‘static’ procedure, which assumes that loads are applied to structures gradually and without dynamic effects, and exported the file as a CAE file, which is a standard file type in the FEA software, Abaqus.

Once the CAE file was imported into Abaqus (Simulia), we applied muscle forces to the models to accurately assess the effects of muscle loading on the skull during a feeding simulation. Locations of muscle insertions were derived from Holliday,^106^ Gignac & Erickson,^26^ and Rowe and Snively^44^ (Figure S3). Muscle forces for FMNH PR 2081 were derived from Rowe and Snively,^44^ which were derived from Gignac and Erickson.^26^ Muscle forces for other tyrannosauroids were scaled from FMNH PR 2081 using the subtemporal fenestra method outlined in Sakamoto.^107^ This was done by measuring the surface area of the adductor chamber using ImageJ and multiplying the surface area by the isometric muscle tension of 31.5 N/cm^2^. This method has proved to be a reliable proxy across amniote clades; additionally, most biomechanical modeling of the amniote skull reliably falls within a predictive distribution with no theropod dinosaur taxa as outliers.^108^ We scaled these values using the surface area measurements of each model and the muscle force components to test for the effects of only skull shape in our second set of analyses (Table S4).

Muscle insertions were applied by selecting nodes along the insertion area. The muscle force components were divided by the number of nodes selected for that muscle, and the resultant forces were applied at X, Y, Z. When muscle insertions and forces were all accounted for, we selected the Create Job tool in Abaqus/CAE 6.14-1 and submitted the request. Once the analysis was finished, we exported von Mises stresses, max strain, and element volume spreadsheets for each model. Because the models were a mix of CT scans and surface scans and we were comparing their biomechanical output, we calculated the mesh-weighted arithmetic mean (MWAM) in RStudio.^109^ This method accounts for element size differences within non-uniform meshes and has been used in previous biomechanical studies^110^^,^^111^^,^^112^; it reduces discrepancies in von Mises stress between 3D models derived from CT scans and surface scans.

We scaled muscle forces in each tyrannosauroid model by measuring surface area values of each model in Avizo Lite 9.5 and MeshLab 2020.06. We scaled each cranium model relative to FMNH PR 2081’s cranium, and each mandible model relative to FMNH PR 2081’s mandible by adjusting muscle force components based on surface area values accordingly in each Abaqus database file. We then reran each FE analysis using the same assumptions and calculated the MWAM. We recorded element stress and strain. Stress is the force per unit area, and one possible measurement of how close a structure is to breakage, which is typically expressed in Mega Pascals (MPa). Strain is the unitless measurement of both how much deformation occurs in the structure and another possible measurement of how close it is to breakage. We recorded von Mises stress and maximum principal strain-based metrics. The latter have been suggested to better describe and predict the mechanical behaviour of bone than stress-based metrics.^113^ We then calculated the mesh-weighted von Mises stress.^110^^,^^111^^,^^112^

#### Muscle sensitivity analysis

We applied muscle sensitivity analyses after calculating results from the actual sized and muscle force scaled models. While previous studies have shown good predictive power of the subtemporal fenestrae and skull width in determining bite forces,^114^ this step was important due to the number of assumptions necessary when studying the musculature of extinct taxa, including muscle placement and size.^115^ This was done by using Python scripts to generate muscle force components 10% higher and lower than the initial values for each individual muscle group and reran multiple analyses.^111^ We selected Macro Manager in Abaqus/CAE 6.14-1 and selected Work under the directory where the other files were stored. The macro would then begin recording our actions. We applied the 10% increases and decreases across the muscle force components of the *Raptorex*, *Albertosaurus,* and largest *Tyrannosaurus* crania and mandibles, submitted the job request, and then stopped recording the macro. In Notepad, the resultant Python script was opened and edited to remove the first line which defined the name of the script and any blank spaces before the start of other lines. The file was saved and copied across all other tyrannosauroid folders containing CAE files.

### Quantification and statistical analysis

No statistical analyses were performed in this study.

## Supporting citations

The following references appear in the Supplemental information: ^68^^,^^69^^,^^70^.

## References

[1] Osborn H.F. (1905). *Tyrannosaurus* and other Cretaceous carnivorous dinosaurs. Bull. Am. Mus. Nat. Hist..

[2] Osborn H.F. (1906). *Tyrannosaurus*, Upper Cretaceous carnivorous dinosaur (second communication). Bull. Am. Mus. Nat. Hist..

[3] Russell D.A. (1970). Tyrannosaurs from the Late Cretaceous of Western Canada. Nat. Mus. Nat. Sci. Pub. Paleontol..

[4] Smith J.B. (2005). Heterodonty in *Tyrannosaurus rex*: Implications for the taxonomic and systematic utility of theropod dentitions. J. Vertebrate Paleontol..

[5] Stevens K.A., Larson P., Wills E.D., Anderson A., Larson P., Carpenter K. (2008). Tyrannosaurus rex: The Tyrant King. Book Publishers.

[6] Xu X., Norell M.A., Kuang X., Wang X., Zhao Q., Jia C. (2004). Basal tyrannosauroids from China and evidence for protofeathers in tyrannosauroids. Nature.

[7] Xu X., Clark J.M., Forster C.A., Norell M.A., Erickson G.M., Eberth D.A., Jia C., Zhao Q. (2006). A basal tyrannosauroid dinosaur from the Late Jurassic of China. Nature.

[8] Brusatte S.L., Carr T.D. (2016). The phylogeny and evolutionary history of tyrannosauroid dinosaurs. Sci. Rep..

[9] Persons W.S., Currie P.J., Erickson G.M. (2020). An older and exceptionally large adult specimen of *Tyrannosaurus rex*. Anat. Rec..

[10] Carr T.D. (1999). Craniofacial ontogeny in Tyrannosauridae (Dinosauria, Coelurosauria). J. Vertebr. Paleontol..

[11] Carr T.D. (2020). A high-resolution growth series of *Tyrannosaurus rex* obtained from multiple lines of evidence. PeerJ.

[12] Longrich N.R., Saitta E.T. (2024). Taxonomic status of *Nanotyrannus lancensis* (Dinosauria: Tyrannosauroidea)—A distinct taxon of small-bodied tyrannosaur. Fossil Stud..

[13] Blanckenhorn W.U. (2000). The evolution of body size: what keeps organisms small?. Q. Rev. Biol..

[14] Therrien F., Henderson D.M. (2007). My theropod is bigger than yours... or not: Estimating body size from skull length in theropods. J. Vertebr. Paleontol..

[15] Sander P.M., Christian A., Clauss M., Fechner R., Gee C.T., Griebeler E.-M., Gunga H.-C., Hummel J., Mallison H., Perry S.F. (2011). Biology of the sauropod dinosaurs: the evolution of gigantism. Biol. Rev. Camb. Philos. Soc..

[16] Bates K.T., Mannion P.D., Falkingham P.L., Brusatte S.L., Hutchinson J.R., Otero A., Sellers W.I., Sullivan C., Stevens K.A., Allen V. (2016). Temporal and phylogenetic evolution of the sauropod dinosaur body plan. R. Soc. Open Sci..

[17] Alexander R.M. (1989).

[18] Alexander R.M. (1991). How dinosaurs ran. Sci. Am..

[19] Alexander R.M. (2006). Dinosaur biomechanics. Proc. Biol. Sci..

[20] Bishop P.J., Cuff A.R., Hutchinson J.R. (2021). How to build a dinosaur: Musculoskeletal modeling and simulation of locomotor biomechanics in extinct animals. Paleobiology.

[21] Hutchinson J.R., Garcia M. (2002). *Tyrannosaurus* was not a fast runner. Nature.

[22] Hutchinson J.R., Ng-Thow-Hing V., Anderson F.C. (2007). A 3D interactive method for estimating body segmental parameters in animals: Application to the turning and running performance of *Tyrannosaurus rex*. J. Theor. Biol..

[23] Sellers W.I., Pond S.B., Brassey C.A., Manning P.L., Bates K.T. (2017). Investigating the running abilities of *Tyrannosaurus rex* using stress-constrained multibody dynamic analysis. PeerJ.

[24] Snively E., O’Brien H., Henderson D.M., Mallison H., Surring L.A., Burns M.E., Holtz T.R., Russell A.P., Witmer L.M., Currie P.J. (2019). Lower rotational inertia and larger leg muscles indicate more rapid turns in tyrannosaurids than in other large theropods. PeerJ.

[25] Rayfield E.J., Barrett P.M., Milner A.R. (2011).

[26] Gignac P.M., Erickson G.M. (2017). The biomechanics behind extreme osteophagy in *Tyrannosaurus rex*. Sci. Rep..

[27] Brusatte S.L., Carr T.D., Erickson G.M., Bever G.S., Norell M.A. (2009). A long-snouted, multihorned tyrannosaurid from the Late Cretaceous of Mongolia. Proc. Natl. Acad. Sci. USA.

[28] Ross C.F. (2005). Finite element analysis in vertebrate biomechanics. Anat. Rec. A Discov. Mol. Cell. Evol. Biol..

[29] Rayfield E.J. (2007). Finite element analysis and understanding the biomechanics and evolution of living and fossil organisms. Annu. Rev. Earth Planet Sci..

[30] Pierce S.E., Angielczyk K.D., Rayfield E.J. (2009). Shape and mechanics in thalattosuchian (Crocodylomorpha) skulls: implications for feeding behaviour and niche partitioning. J. Anat..

[31] Morales-García N., Gill P., Janis C., Rayfield E.J. (2021). Jaw shape and mechanical advantage are indicative of diet in Mesozoic mammals. Comm. Biol..

[32] Rayfield E.J. (2005). Aspects of comparative cranial mechanics in the theropod dinosaurs *Coelophysis, Allosaurus* and *Tyrannosaurus*. Zool. J. Linn. Soc..

[33] Ma W., Pittman M., Butler R.J., Lautenschlager S. (2022). Macroevolutionary trends in theropod dinosaur feeding mechanics. Curr. Biol..

[34] Bell P.R., Snively E., Shychoski L. (2009). A comparison of the jaw mechanics in hadrosaurid and ceratopsid dinosaurs using finite element analysis. Anat. Rec..

[35] Lautenschlager S., Witmer L.M., Altangerel P., Rayfield E.J. (2013). Edentulism, beaks, and biomechanical innovations in the evolution of theropod dinosaurs. Proc. Natl. Acad. Sci. USA.

[36] Lautenschlager S., Butler R.J. (2016). Neural and endocranial anatomy of Triassic phytosaurian reptiles and convergence with fossil and modern crocodylians. PeerJ.

[37] Cost I.N., Middleton K.M., Sellers K.C., Echols M.S., Witmer L.M., Davis J.L., Holliday C.M. (2020). Palatal biomechanics and its significance for cranial kinesis in *Tyrannosaurus rex*. Anat. Rec..

[38] Barbosa G.G., Langer M.C., de Oliveira Martins N., Montefeltro F.C. (2023). Assessing the palaeobiology of *Vespersaurus paranaensis* (Theropoda, Noasauridae), Cretaceous, Bauru Basin – Brazil, using Finite Element Analysis. Cretac. Res..

[39] Johnson-Ransom E., Li F., Xu X., Ramos R., Midzuk A.J., Thon U., Atkins-Weltman K., Snively E. (2023). Comparative cranial biomechanics reveal that Late Cretaceous tyrannosaurids exerted relatively greater bite force than in early-diverging tyrannosauroids. Anat. Rec..

[40] Reims N., Böhnel M., Larson P., Schulp A. (2016). 19th World Conference on Non-Destructive Testing.

[41] Cunningham J.A., Rahman I.A., Lautenschlager S., Rayfield E.J., Donoghue P.C.J. (2014). A virtual world of paleontology. Trends Ecol. Evol..

[42] Díez Díaz V., Mallison H., Asbach P., Schwarz D., Blanco A. (2021). Comparing surface digitization techniques in palaeontology using visual perceptual metrics and distance computations between 3D meshes. Palaeontology.

[43] Rowe A.J., Rayfield E.J. (2022). The efficacy of computed tomography scanning versus surface scanning in 3D finite element analysis. PeerJ.

[44] Rowe A.J., Snively E. (2022). Biomechanics of juvenile tyrannosaurid mandibles and their implications for bite force: Evolutionary biology. Anat. Rec..

[45] Reichel M. (2012). The variation of angles between anterior and posterior carinae of tyrannosaurid teeth. Can. J. Earth Sci..

[46] Brusatte S.L., Norell M.A., Carr T.D., Erickson G.M., Hutchinson J.R., Balanoff A.M., Bever G.S., Choiniere J.N., Makovicky P.J., Xu X. (2010). Tyrannosaur paleobiology: New research on ancient exemplar organisms. Science.

[47] Chin K., Tokaryk T.T., Erickson G.M., Calk L.C. (1998). A king-sized theropod coprolite. Nature.

[48] DePalma R.A., Burnham D.A., Martin L.D., Rothschild B.M., Larson P.L. (2013). Physical evidence of predatory behavior in *Tyrannosaurus rex*. Proc. Natl. Acad. Sci. USA.

[49] Foffa D., Cuff A.R., Sassoon J., Rayfield E.J., Mavrogordato M.N., Benton M.J. (2014). Functional anatomy and feeding biomechanics of a giant upper Jurassic pliosaur (Reptilia: Sauropterygia) from Weymouth Bay, Dorset, UK. J. Anat..

[50] Foster W., Brusatte S.L., Carr T.D., Williamson T.E., Yi L., Lü J. (2022). The cranial anatomy of the long-snouted tyrannosaurid dinosaur *Qianzhousaurus sinensis* from the Upper Cretaceous of China. J. Vertebr. Paleontol..

[51] Bell P.R., Currie P.J., Lee Y.N. (2012). Tyrannosaur feeding traces on *Deinocheirus* (Theropoda:?Ornithomimosauria) remains from the Nemegt Formation (Late Cretaceous), Mongolia. Cretac. Res..

[52] Owocki K., Kremer B., Cotte M., Bocherens H. (2020). Diet preferences and climate inferred from oxygen and carbon isotopes of tooth enamel of *Tarbosaurus bataar* (Nemegt Formation, Upper Cretaceous, Mongolia). Palaeogeogr. Palaeoclimatol. Palaeoecol..

[53] Hone D.W., Watabe M. (2010). New information on scavenging and selective feeding behaviour of tyrannosaurids. Acta Palaeontol. Pol..

[54] Borsuk-Bialynicka M. (1977). A new camarasaurid sauropod *Opisthocoelicaudia skarzynskii* gen. n., sp. n. from the Upper Cretaceous of Mongolia. Palaeontol. Pol..

[55] Fowler D.W., Sullivan R.M. (2006). A ceratopsid pelvis with toothmarks from the Upper Cretaceous Kirtland Formation, New Mexico: Evidence of Late Campanian tyrannosaurids feeding behavior. New Mex. Mus. Nat. Hist. Sci. Bull..

[56] Holtz T.R., Larson P., Carpenter K. (2008). *Tyrannosaurus rex*: The Tyrant King. Book Publishers.

[57] Erickson G.M., Olson K.H. (1996). Bite marks attributable to *Tyrannosaurus rex*: Preliminary description and implications. J. Vertebr. Paleontol..

[58] Ruxton G.D., Houston D.C. (2003). Could *Tyrannosaurus rex* have been a scavenger rather than a predator? An energetics approach. Proc. Biol. Sci..

[59] Herbers J.M. (1981). Time resources and laziness in animals. Oecologia.

[60] Kane A., Healy K., Ruxton G.D., Jackson A.L. (2016). Body size as a driver of scavenging in theropod dinosaurs. Am. Nat..

[61] Carpenter K. (1998). Evidence of predatory behavior by carnivorous dinosaurs. GAIA Ecol. Perspect. Sci. Soc..

[62] Persons W.S., Currie P.J., Eberth D.A., Evans D.C. (2014). Hadrosaurs.

[63] Li D., Norell M.A., Gao K.Q., Smith N.D., Makovicky P.J. (2010). A longirostrine tyrannosauroid from the Early Cretaceous of China. Proc. Biol. Sci..

[64] Lü J., Yi L., Brusatte S.L., Yang L., Li H., Chen L. (2014). A new clade of Asian Late Cretaceous long-snouted tyrannosaurids. Nat. Commun..

[65] Loewen M.A., Irmis R.B., Sertich J.J.W., Currie P.J., Sampson S.D. (2013). Tyrant dinosaur evolution tracks the rise and fall of Late Cretaceous oceans. PLoS One.

[66] Lambe L.M. (1914). On a new genus and species of carnivorous dinosaur from the Belly River Formation of Alberta, with a description of the skull of *Stephanosaurus marginatus* from the same horizon. Ottawa Nat..

[67] Carr T.D., Varricchio D.J., Sedlmayr J.C., Roberts E.M., Moore J.R. (2017). A new tyrannosaur with evidence for anagenesis and crocodile-like facial sensory system. Sci. Rep..

[68] Currie P.J. (2003). Cranial anatomy of tyrannosaurid dinosaurs from the Late Cretaceous of Alberta, Canada. Acta Palaeontol. Pol..

[69] Padian K. (2022). Why tyrannosaurid forelimbs were so short: An integrative hypothesis. Acta Palaeontol. Pol..

[70] Christiansen P., Fariña † R.A. (2004). Mass prediction in theropod dinosaurs. Hist. Biol..

[71] Campione N.E., Evans D.C., Brown C.M., Carrano M.T. (2014). Body mass estimation in non-avian bipeds using a theoretical conversion to quadruped stylopodial proportions. Methods Ecol. Evol..

[72] Carr T.D., Williamson T.E. (2010). *Bistahieversor sealeyi*, gen. et sp. Nov., a new tyrannosauroid from New Mexico and the origin of deep snouts in Tyrannosauroidea. J. Vertebr. Paleontol..

[73] Molina-Perez R., Larramendi A. (2019).

[74] McKeown M., Brusatte S.L., Williamson T.E., Schwab J.A., Carr T.D., Butler I.B., Muir A., Schroeder K., Espy M.A., Hunter J.F. (2020). Neurosensory and sinus evolution as tyrannosauroid dinosaurs developed giant size: Insight from the endocranial anatomy of *Bistahieversor sealeyi*. Anat. Rec..

[75] Bates K.T., Falkingham P.L. (2012). Estimating maximum bite performance in *Tyrannosaurus rex* using multi-body dynamics. Biol. Lett..

[76] Currie P.J. (1998). Possible evidence of gregarious behaviour in tyrannosaurids. Gaia.

[77] Eberth D.A., Currie P.J. (2010). Stratigraphy, sedimentology, and taphonomy of the *Albertosaurus* bonebed (upper Horseshoe Canyon Formation; Maastrichtian), southern Alberta, Canada. Can. J. Earth Sci..

[78] Coppock C.C., Currie P.J. (2024). Additional *Albertosaurus sarcophagus* (Tyrannosauridae, Albertosaurinae) material from the Danek Bonebed of Edmonton, Alberta, Canada with evidence of cannibalism. Can. J. Earth Sci..

[79] Tanke D., Currie P.J. (1998). Head-biting behavior in theropod dinosaurs: Paleopathological evidence. Gaia.

[80] Brown C.M., Currie P.J., Therrien F. (2022). Intraspecific facial bite marks in tyrannosaurids provide insight into sexual maturity and evolution of bird-like intersexual display. Paleobiology.

[81] Bell P.R., Currie P.J. (2010). A tyrannosaur jaw bitten by a confamilial: scavenging or fatal agonism?. Lethaia.

[82] Hone D.W.E., Tanke D.H. (2015). Pre- and postmortem tyrannosaurid bite marks on the remains of *Daspletosaurus* (Tyrannosaurinae: Theropoda) from Dinosaur Provincial Park, Alberta, Canada. PeerJ.

[83] Currie P.J., Trexler D., Koppelhus E.B., Wicks K., Murphy N., Carpenter K. (2005). The Carnivorous Dinosaurs.

[84] Eberth D.A., McCrea R.T. (2001). Were large theropods gregarious?. J. Vertebr. Paleontol..

[85] Kurzanov S.M. (1976). A new carnosaur from the Late Cretaceous of Nogon-Tsav, Mongolia. Joint Soviet-Mongolian Paleontol. Expedition Trans..

[86] Sereno P.C., Tan L., Brusatte S.L., Kriegstein H.J., Zhao X., Cloward K. (2009). Tyrannosaurid skeletal design first evolved at small body size. Science.

[87] Fowler D.W., Woodward H.N., Freedman E.A., Larson P.L., Horner J.R. (2011). Reanalysis of “*Raptorex kriegsteini*”: A juvenile tyrannosaurid dinosaur from Mongolia. PLoS One.

[88] Maleev E.A. (1955). Translated by F. J. Alcock. New carnivorous dinosaurs from the Upper Cretaceous of Mongolia. Dokl. Akad. Nauk SSSR.

[89] Carr T.D. (2023). A reappraisal of tyrannosauroid fossils from the Iren Dabasu Formation (Coniacian–Campanian), Inner Mongolia, People’s Republic of China. J. Vertebr. Paleontol..

[90] Brochu C.A. (2003). Osteology of *Tyrannosaurus rex*: Insights from a nearly complete skeleton and high-resolution computed tomographic analysis of the skull. J. Vertebrate Paleontol..

[91] Holtz T.R., Weishampel D.B., Dodson P., Osmólska H. (2004). The Dinosauria.

[92] Lautenschlager S. (2015). Estimating cranial musculoskeletal constraints in theropod dinosaurs. R. Soc. Open Sci..

[93] Snively E., Henderson D.M., Phillips D.S. (2006). Fused and vaulted nasals of tyrannosaurid dinosaurs: Implications for cranial strength and feeding mechanics. Acta Palaeontol. Pol..

[94] Rayfield E.J. (2004). Cranial mechanics and feeding in *Tyrannosaurus rex*. Proc. Biol. Sci..

[95] Snively E., Russell A.P. (2007). Functional variation of neck muscles and their relation to feeding style in Tyrannosauridae and other large theropod dinosaurs. Anat. Rec..

[96] Longrich N.R., Horner J.R., Erickson G.M., Currie P.J. (2010). Cannibalism in *Tyrannosaurus rex*. PLoS One.

[97] Anderson J.S. (1999). Occipital condyle in the ceratopsian dinosaur *Triceratops*, with comments on body size variation. Contrib. Mus. Paleontol. Univ. Mich..

[98] Engelman R.K. (2022). Occipital condyle width (OCW) is a highly accurate predictor of body mass in therian mammals. BMC Biol..

[99] Gold M.E.L., Brusatte S.L., Norell M.A. (2013). The cranial pneumatic sinuses of the tyrannosaurid *Alioramus* (Dinosauria: Theropoda) and the evolution of cranial pneumaticity in theropod dinosaurs. Am. Mus. Novit..

[100] Kammerer C.F., Deutsch M., Lungmus J.K., Angielczyk K.D. (2020). Effects of taphonomic deformation on geometric morphometric analysis of fossils: a study using the dicynodont *Diictodon feliceps* (Therapsida, Anomodontia). PeerJ.

[101] Demuth O.E., Benito J., Tschopp E., Lautenschlager S., Mallison H., Heeb N., Field D.J. (2022). Topology-based three-dimensional reconstruction of delicate skeletal fossil remains and the quantification of their taphonomic deformation. Front. Ecol. Evol..

[102] Zapata U., Metzger K., Wang Q., Elsey R.M., Ross C.F., Dechow P.C. (2010). Material properties of mandibular cortical bone in the American alligator, *Alligator mississippiensis*. Bone.

[103] Porro L.B., Holliday C.M., Anapol F., Ontiveros L.C., Ontiveros L.T., Ross C.F. (2011). Free body analysis, beam mechanics, and finite element modeling of the mandible of *Alligator mississippiensis*. J. Morphol..

[104] Ballell A., Moon B.C., Porro L.B., Benton M.J., Rayfield E.J. (2019). Convergence and functional evolution of longirostry in crocodylomorphs. Palaeontology.

[105] Herbst E.C., Lautenschlager S., Bastiaans D., Miedema F., Scheyer T.M. (2021). Modeling tooth enamel in FEA comparisons of skulls: Comparing common simplifications with biologically realistic models. iScience.

[106] Holliday C.M. (2009). New insights into dinosaur jaw muscle anatomy. Anat. Rec..

[107] Sakamoto M. (2006). Scaling bite force in predatory animals: How does *T. rex* compare with living predators?. J. Vertebr. Paleontol..

[108] Sakamoto M. (2021). Assessing bite force estimates in extinct mammals and archosaurs using phylogenetic predictions. Palaeontology.

[109] R Core Team (2021). https://www.R-project.org/.

[110] Marcé-Nogué J., de Esteban-Trivigno S., Escrig C., Gil L. (2016). Accounting for differences in element size and homogeneity when comparing Finite Element models: Armadillos as a case study. Palaeontol. Electron..

[111] Morales-García N.M., Burgess T.D., Hill J.J., Gill P.G., Rayfield E.J. (2019). The use of extruded finite-element models as a novel alternative to tomography-based models: a case study using early mammal jaws. J. R. Soc. Interface.

[112] Ballell A., Ferrón H.G. (2021). Biomechanical insights into the dentition of megatooth sharks (Lamniformes: Otodontidae). Sci. Rep..

[113] Dutel H., Gröning F., Sharp A.C., Watson P.J., Herrel A., Ross C.F., Jones M.E.H., Evans S.E., Fagan M.J. (2021). Comparative cranial biomechanics in two lizard species: impact of variation in cranial design. J. Experi. Biol..

[114] Sakamoto M. (2022). Estimating bite force in extinct dinosaurs using phylogenetically predicted physiological cross-sectional areas of jaw adductor muscles. PeerJ.

[115] Bates K.T., Falkingham P.L. (2018). The importance of muscle architecture in biomechanical reconstructions of extinct animals: a case study using *Tyrannosaurus rex*. J. Anat..

