# Supplementary material for: Morphological evolution and functional consequences of giantism in tyrannosauroid dinosaurs
Source: iScience. 2024 Aug 5;27(9):110679. doi: 10.1016/j.isci.2024.110679 (PMC11387897; doi:10.1016/j.isci.2024.110679)
Supplement: Document S1. Figures S1–S3 and Tables S1–S4 [file mmc1.pdf]

iScience, Volume *27*

Supplemental information

Morphological evolution and functional consequences of giantism in tyrannosauroid dinosaurs

Andre J. Rowe and Emily J. Rayfield

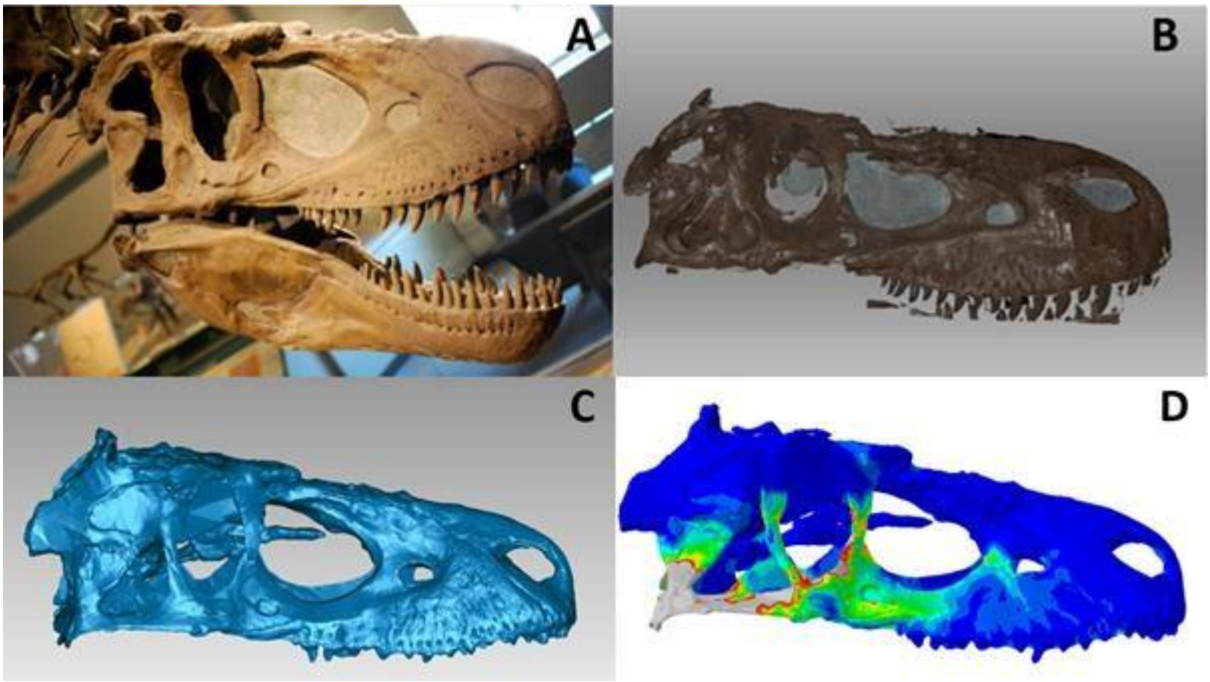

Figure S1. 3D finite element analysis workflow demonstrated using the *Daspletosaurus torosus* skull, related to STAR Methods and Figure 2.

(A) FMNH PR308 skull replica mounted at the Field Museum, Chicago, IL, USA; (B) aligned surface scans of FMNH PR308 in Artec Studio Professional 14 prior to Sharp Fusion, which converts scans into the STL file type; (C) STL file in Geomagic Studio 12; (D) finite element model in Abaqus/CAE 6.14-1.

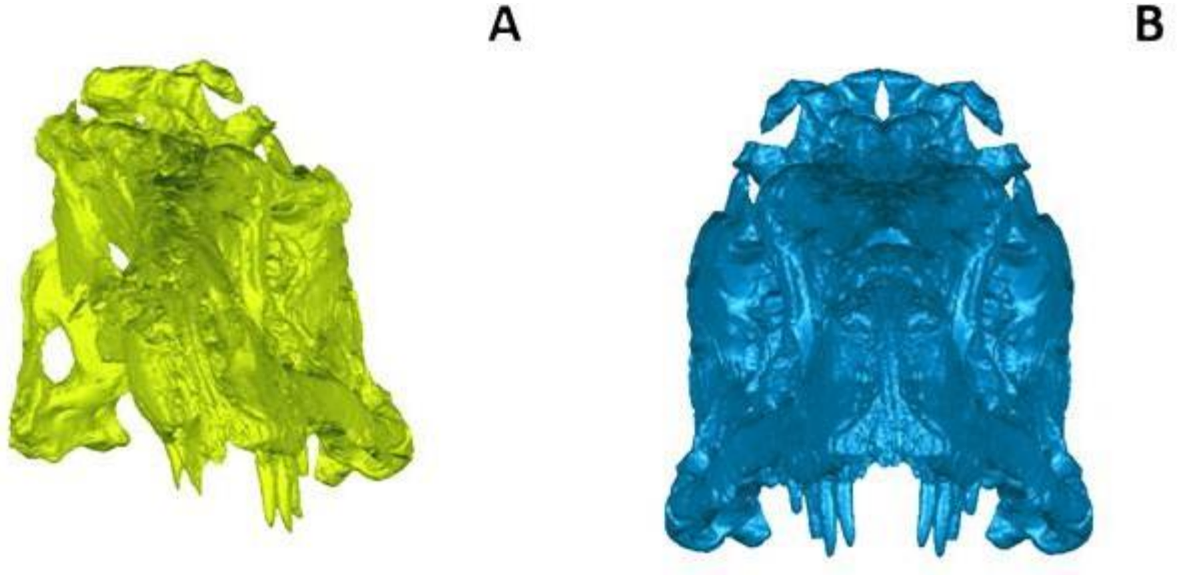

Figure S2. *Bistahieversor sealeyi* 3D cranial model used in this study, related to STAR Methods.

(A) Deformed *Bistahieversor* cranium in anterior view, and (B) *Bistahieversor* cranium after digital retrodeformation in Artec Studio Professional 14, MeshLab 2020.06, and Geomagic Studio 12.

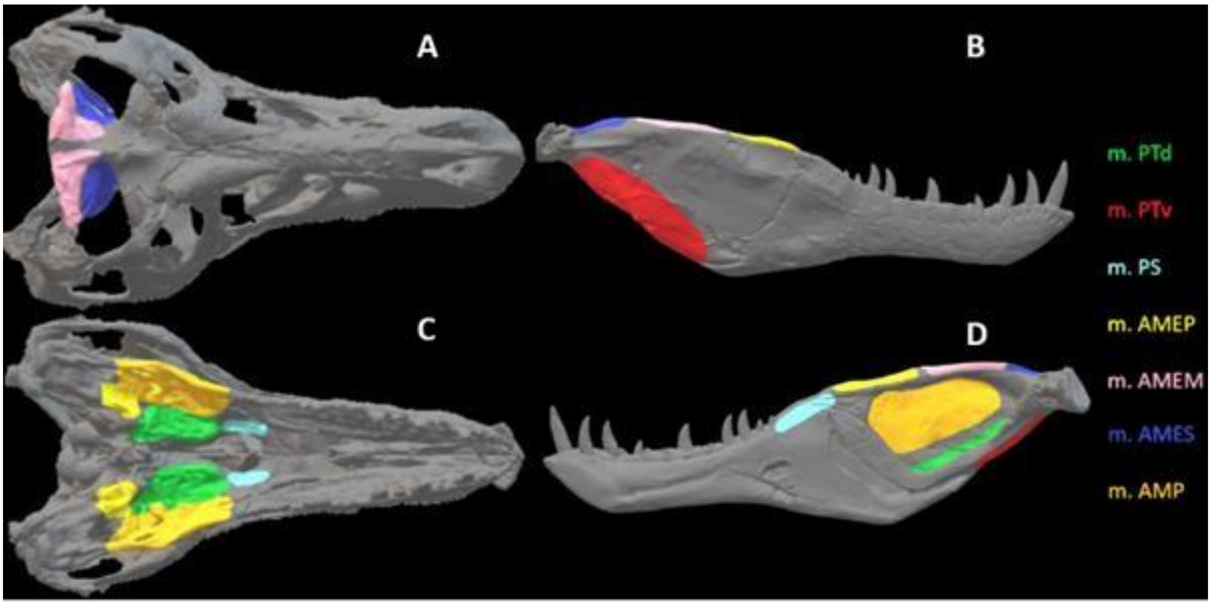

Figure S3. ***Tyrannosaurus rex*** **(USNM 555000) skull model illustrating node placement to simulate muscle attachment sites, related to STAR Methods and Figures 2 and 4.**

(A) Cranium in dorsal view; (B) mandible in lateral view; (C) cranium in ventral view; (D) mandible in medial view. Muscle abbreviations: m. PTd, M. pterygoideus dorsalis; m. PTv, M. pterygoideus ventralis; m. PS, M. pseudotemporalis complex; m. AMEP, M. adductor mandibulae externus profundus; m. AMEM, Musculus adductor mandibulae externus medialis; m. AMES, M. adductor mandibulae externus superficialis; m. AMP, M. adductor mandibulae posterior.

Table S1. Specimen numbers for each tyrannosauroid specimen included in this study, related to STAR Methods.

Specimen name	Specimen number	Skull length from literature (mm)	Skull length from MeshLab (mm)	Occipital condyle diameter (mm)
Raptorex kriegsteini	LH PV18	300 (Sereno et al.[83])	296	8
Bistahieversor sealeyi	NMMNH P- 27469	1,070 (Carr & Williamson[69])	902	45
Albertosaurus sarcophagus	TMP 1981.010.0001	880 (Currie[113])	768	52
Alioramus altai	IGM 100/1844	700 (Lü et al.[64])	527	23
Daspletosaurus torosus	FMNH PR308	1,050 (Carr[11])	969	66
Tyrannosaurus rex	USNM 555000	1,200 (Padian[114])	1, 285	106
Tyrannosaurus rex	FMNH PR 2081	1,275 (Gignac & Erickson[27])	1, 290	110

Table S2. Body mass estimates from the literature (kg) and ontogenetic stages/chronological ages where available from the literature, related to STAR methods.

Specimen name	Specimen number	Body mass estimate (kg)	Ontogenetic stage/chronological age
Raptorex kriegsteini	LH PV18	65 (Sereno et al.[83])	Juvenile; 3-6 years old (Fowler et al.[84])
Bistahieversor sealeyi	NMMNH P-27469	3,300 (Molina-Pérez & Larramendi[70])	Adult
Albertosaurus sarcophagus	TMP 1981.010.0001	1,685 (Christiansen & Farina[115])	Adult
Alioramus altai	IGM 100/1844	369 (Brusatte et al.[28])	Juvenile; 9 years old (Brusatte et al.[28])
Daspletosaurus torosus	FMNH PR308	2,700 (Christiansen & Farina[115])	Adult
Tyrannosaurus rex	USNM 555000	6,216 (Campione et al.[68])	Adult; 23-27 years old (Carr[12])
Tyrannosaurus rex	FMNH PR 2081	7,377 (Campione et al.[68])	Senescent adult; 28 years old (Carr[12])

Table S3. Quantitative properties for each 3D mesh which were calculated in MeshLab 2020.06, related to STAR Methods.

Specimen name	Cranial triangle count	Mandibular triangle count	Cranial element counts	Mandibular element counts	Cranial volume (mm^3)	Mandibular volume (mm^3)
Raptorex kriegsteini	289,478	204,770	434,217	307,155	144,711	102,385
Bistahieversor sealeyi	923,682	529,002	1,385,523	793,503	461,821	264,485
Albertosaurus sarcophagus	651,876	317,422	977,814	476,133	325,886	158,705
Alioramus altai	465,994	38,102	698,991	57,153	23,296	19,045
Daspletosaurus torosus	386,920	467,596	580,380	956,394	193,440	318,802
Tyrannosaurus rex (USNM 555000)	1,270,954	711,910	1,906,431	1,067,865	635,435	355,957
Tyrannosaurus rex (FMNH PR 2081)	1,284,114	860,746	1,926,171	1,291,119	642,017	430,375

Table S4. Surface area values for each model which were calculated in both MeshLab 2020.06 and Avizo Lite 9.5 to check for consistency, related to STAR Methods and Figures 4 and 5.

Specimen name	Specimen number	Cranial model surface area (mm^2)	Mandible model surface area (mm^2)
Raptorex kriegsteini	LH PV18	904346	49811
Bistahieversor sealeyi	NMMNH P-27469	1777871	885024
Albertosaurus sarcophagus	TMP 1981.010.0001	1382871	677021
Alioramus altai	IGM 100/1844	457843	160486
Daspletosaurus torosus	FMNH PR308	1526939	803412
Tyrannosaurus rex	USNM 555000	4600059	1921720
Tyrannosaurus rex	FMNH PR 2081	4604695	1924344
